# Near-Unity Photoluminescence Quantum Yield of Core-Only InP Quantum Dots *via* a Simple Postsynthetic InF_3_ Treatment

**DOI:** 10.1021/acsnano.4c03290

**Published:** 2024-05-22

**Authors:** Maarten Stam, Guilherme Almeida, Reinout F. Ubbink, Lara M. van der Poll, Yan B. Vogel, Hua Chen, Luca Giordano, Pieter Schiettecatte, Zeger Hens, Arjan J. Houtepen

**Affiliations:** † Optoelectronic Materials Section, Faculty of Applied Sciences, Delft University of Technology, Van der Maasweg 9, 2629 HZ Delft, The Netherlands; ‡ Physics and Chemistry of Nanostructures, Department of Chemistry, Ghent University, 9000 Gent, Belgium

**Keywords:** quantum dots, InP, near-unity quantum yield, photoluminescence, phosphors

## Abstract

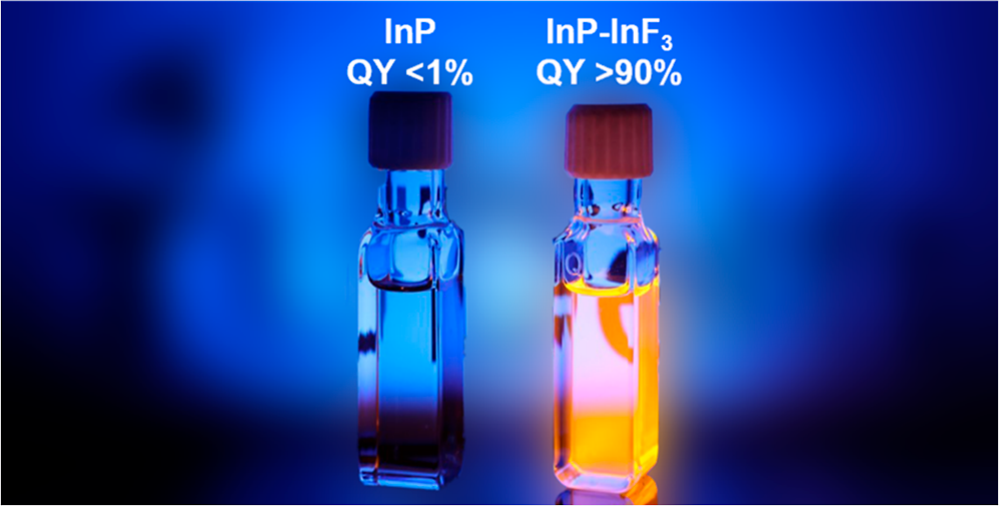

Indium phosphide (InP) quantum dots (QDs) are considered the most promising alternative for Cd and Pb-based QDs for lighting and display applications. However, while core-only QDs of CdSe and CdTe have been prepared with near-unity photoluminescence quantum yield (PLQY), this is not yet achieved for InP QDs. Treatments with HF have been used to boost the PLQY of InP core-only QDs up to 85%. However, HF etches the QDs, causing loss of material and broadening of the optical features. Here, we present a simple postsynthesis HF-free treatment that is based on passivating the surface of the InP QDs with InF_3._ For optimized conditions, this results in a PLQY as high as 93% and nearly monoexponential photoluminescence decay. Etching of the particle surface is entirely avoided if the treatment is performed under stringent acid-free conditions. We show that this treatment is applicable to InP QDs with various sizes and InP QDs obtained *via* different synthesis routes. The optical properties of the resulting core-only InP QDs are on par with InP/ZnSe/ZnS core–shell QDs, with significantly higher absorption coefficients in the blue, and with potential for faster charge transport. These are important advantages when considering InP QDs for use in micro-LEDs or photodetectors.

## Introduction

Luminescent materials are of great importance in daily life applications such as displays and lighting. Colloidal quantum dots (QDs) are a unique class of luminescent materials with their high photoluminescence (PL) quantum yield (PLQY), narrow emission, and size-dependent optical properties. These qualities make QDs promising candidates for application in, *e.g.*, light-emitting diodes, photodetectors, biomedical imaging, lasers, and photovoltaics.^1−13^ In particular, InP-based QDs are of commercial interest since the material is free of toxic and RoHS-restricted elements such as Cd and Pb.^14,15^

In general, the as-synthesized InP QDs cores have a PLQY lower than 1% and show significant trap state emission.^16−20^ It has been discussed that PL quenching is, to a significant amount, caused by surface oxides and undercoordinated P atoms on the surface.^18,21,22^ Surface oxides are often removed with HF, either added directly or formed *in situ* in a postsynthesis treatment.^6,18,23,24^ Subsequent growth of wider band gap ZnSe and ZnS shells has resulted in InP/ZnSe/ZnS core/shell/shell QDs with near-unity PLQYs.^6,13,18,24−27^

Synthesizing high PLQY core-only QDs is relevant to fully understand and control how the surface of the QDs affects the optical properties. More practically, high PLQY core-only QDs offer several significant advantages. For example, the synthesis of core-only QDs is, compared to core/shell QDs, relatively simple and requires less material, reducing the production costs. Core-only InP QDs also have a larger absorption coefficient than core/shell QDs at photon energies below the band gap of the shell, implying that thinner layers suffice to absorb a predefined fraction of incoming light. This aspect becomes especially relevant when using QDs as down conversion phosphors in micro-LEDs. Furthermore, the absence of shells is advantageous for charge transport in QD LEDs and photodetectors.^18,28^

The PLQY of core-only InP QDs has been promoted by treating the QDs with aqueous HF. In 1996, it was reported that treatment of InP QDs with HF or NH_4_F resulted in a PLQY of 30%.^29^ It was proposed that the F^–^ ion would fill surface P vacancies (*i.e.*, would bind to surface In) and replace oxygen atoms and therefore boost the PLQY. In further studies, PLQYs were reported up to 40% by treating InP QDs with aqueous HF solutions, sometimes in combination with photoirradiation to induce photoetching.^24,30−33^ Different mechanisms are proposed to describe the (photo)etching, including the removal of oxidized P atoms and fluoride passivation of the surface.^34^ Moreover, HF treatment of InP QDs is often used before shell growth, yielding QDs with a near-unity PLQY values.^6^

Recently, we reported a method to form HF *in situ*, allowing to work water-free and significantly reducing the hazards of working with HF.^18,35,36^ This work showed that the *in situ* HF treatment on core-only InP QDs in the presence of excess ligands that coordinate to surface P anions (so-called Z-type ligands^37^) boosts the PLQY up to 85%.^18^ Solid-state nuclear magnetic resonance (ssNMR) results demonstrated that this *in situ* HF treatment breaks up poly phosphates on the surface but does not remove all oxides. In addition, the HF treatment terminates the QD surface with fluoride ions.^18^

A significant downside of HF treatments is that they lead to etching of the surface, probably *via* the formation of PH_3_ and InF_3_. The same is observed for the *in situ* HF treatment described by Ubbink *et al.*(18) HF etching causes significant material losses and broadens the optical features of InP QDs, significantly reducing their usefulness in light-emitting applications that rely on narrow emission. However, the understanding that emerged from these previous studies is that a high PLQY of InP QDs does not necessarily require HF treatments. Rather, a high PLQY can be achieved if too severe surface oxidation, notably the occurrence of polyphosphates, is prevented, and undercoordinated surface atoms are coordinated with fluoride (for In atoms) and InF_3_ for P atoms.^18^ This is consistent with recent results reported by Reiss and co-workers who showed that the *in situ* HF treatment on oxide-free InP QDs works even at room temperature, which allowed them to achieve a PLQY of up to 79%, while minimizing the broadening of the optical features.^36^

For this reason, we sought a direct treatment that does not involve HF but increases the ligand surface coverage of InP QDs that have only a minor degree of oxidation. Common ligands used to treat QDs are metal halides, metal carboxylates, and metal phosphonates. In the case of Cd-chalcogenide QDs, such Z-type ligands lifted the PLQY above 90%.^38−41^ For InP QDs, some Z-type ligand treatments have been explored, with a highest reported PLQY of 54% for InP QDs capped with Cd-oleate ligands.^16,39,42,43^ This comparably low PLQY raises the question whether there are other surface traps on InP QDs that limit the PLQY.

In this work, we developed a straightforward treatment of InP QDs with InF_3_ as Z-type ligand leading to a PLQY up to 93%. We first screened several metal halide salts as suitable Z-type ligands. We found that InF_3_ yields the highest PLQY under the selected reaction conditions, and we therefore selected this salt as the most promising candidate. Next, the treatment is optimized for surface passivation with InF_3_. The passivation of the surface of the InP QDs requires the partial exchange of the surface capping ligand originating from the synthesis, with InF_3_, which was found to be a thermally activated process. The optimization shows that exposing the InP QDs to InF_3_ in hexadecane for 60 min at 180 °C results in a highest PLQY of 93% and nearly single exponent PL decay curves. We show that the presence of (trace amounts of) protons results in surface etching in addition to ligand exchange. However, this can be prevented completely by working under strict acid-free conditions, allowing us to maintain the narrow fwhm of photoluminescence.

Finally, we show that applying the treatment to different sizes of InP QDs and to InP QDs made *via* different synthesis routes invariably improves the PLQY. Purposely oxidized InP QDs also show an increase in PLQY to ∼40% but do not allow to reach near-unity values. We propose that severe surface oxidation impedes the complete coverage of the QD surface with Z-type ligands. The near-unity PLQY obtained for these core-only InP QDs shows that it is possible to completely heal the surface of InP with postsynthetic ligand treatments, a result suggesting that all nonradiative recombination before treatment occurs *via* surface states. The results further demonstrate that small amounts of surface oxidation are not deleterious for the PLQY, rather the presence of undercoordinated P atoms is. Coordinating these with small fluoride-based ligands, most notably InF_3_, is key to achieving a near-unity PLQY.

The simple InF_3_ treatment allows to reach near-unity PLQYs and results in narrow emission, making these InP core-only QDs interesting for applications in lighting, displays, and photodetection.

## Results/Discussion

### Screening of Metal Halide Ligands to Enhance the PLQY of InP QDs

**Figure 1:**
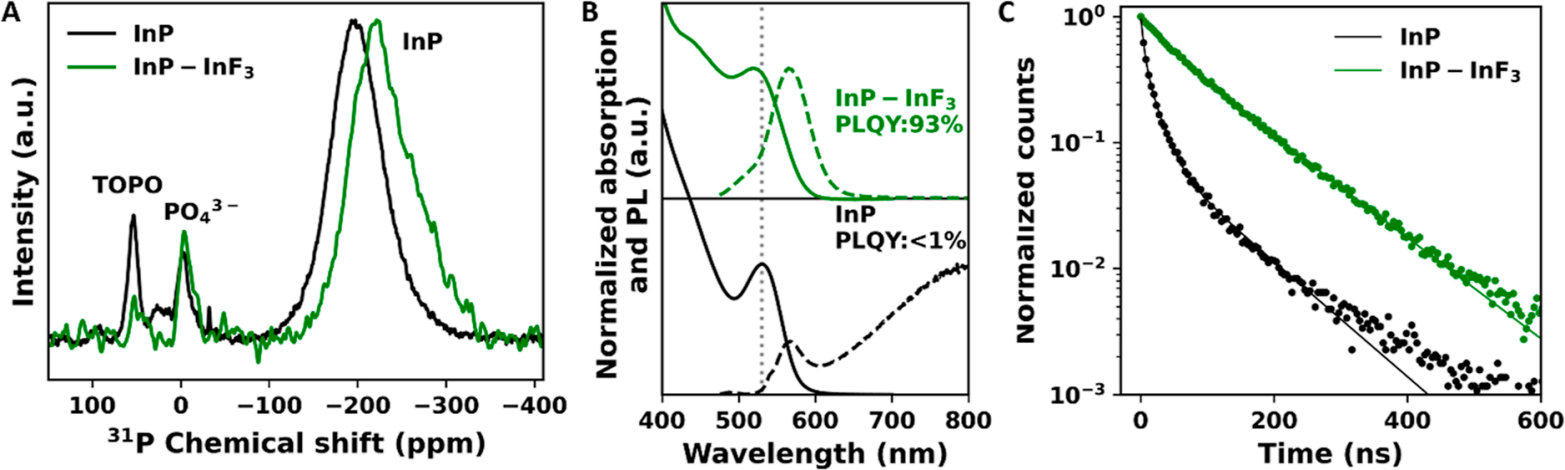
(A) ^31^P ssNMR spectra of InP (black) and InP–InF_3_ (green). (B) Absorption and PL spectra of InP (black) and InP–InF_3_ (green) QDs. The dashed gray line indicates the 1S absorption peak of the InP QDs before treatment. (C) Time-resolved PL lifetime measurements of InP (black) and InP–InF_3_ (green). The solid lines show multiexponential fits to the experimental data (see the text).

Since our previous work indicated that too severe surface oxidation should be avoided to achieve high PLQYs after HF treatment,^18^ we selected a synthesis of InP QDs that minimizes oxidation. The InP QDs used in the first part of this study are made *via* a heat-up synthesis, based on the work of Li *et al.*, as detailed in the Methods/Experimental Section.^44^ This method involves a shorter exposure of In(PA)_3_ to high temperature than typical hot-injection synthesis methods (see the Methods/Experimental Section for details), minimizing the formation of water in an *in situ* condensation reaction of the carboxylic acid ligands, which is known to cause surface oxidation for InP QDs.^45^ The synthesis is performed under an atmosphere of Ar/H_2_ to further minimize the oxidation of the surface of the InP QDs.^46,47^ We find that the degree of surface oxidation is indeed minimized by using this gas mixture and by using a heat-up synthesis *versus* a hot-injection synthesis. A one pulse ^31^P ssNMR spectrum of the InP QDs after synthesis is shown in Figure 1A in black. The resonance belonging to P^3–^ in the InP QDs is present at around −200 ppm, as has been frequently reported.^45,48^ The resonances that are visible at −5 and 55 ppm, respectively, have been assigned to PO_4_^3–^ and trioctylphosphine oxide (TOPO), respectively.^18,49^ TOPO is formed during the synthesis by oxidation of trioctylphosphine (TOP) and coordinates to the surface of the QDs. By integration, we find that ∼5% of the phosphorus atoms of the InP QDs is in the oxidized PO_4_^3–^ state.

**Scheme 1:**
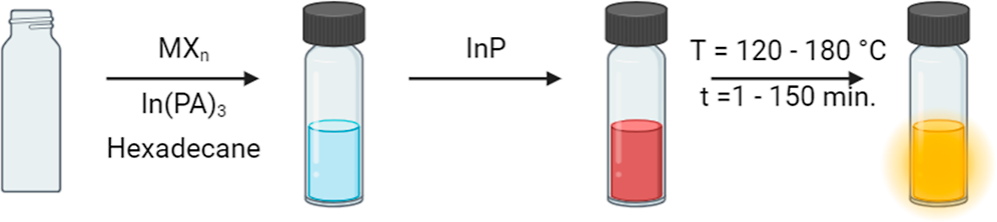
Schematic Description of the InF_3_ Treatment To a vial, hexadecane, In(PA)_3_, and MX_*n*_ are added (with M = Mg, Al, Cd, Zn, and In and X = F, Cl, Br, and I). An InP QD dispersion is added to the solution, and the mixture is heated to the desired temperature for the desired time.

The steady-state absorption and PL spectra of the as-synthesized QDs are shown in Figure 1B using solid and dashed black lines, respectively. These as-synthesized QDs have a PLQY of <1%, on par with the reported values in literature.^16−19^ These QDs exhibit multiexponential PL decay, as shown in Figure 1C (the black solid line is a triexponential fit to the data), with an intensity averaged lifetime of 53 ns. We attempted to increase the PLQY of these QDs using various metal halides as ligands with the procedure that is illustrated in Scheme 1. Briefly, a solid metal halide salt is dispersed in hexadecane together with solid In(PA)_3_ in a vial. In(PA)_3_ is added to maintain colloidal stability, as discussed further below. Then, InP QDs, dispersed in hexadecane, are added, and the mixture is heated to a temperature between 120 and 180 °C for 1 to 150 min. The green spectra in Figure 1B, the green PL transient in Figure 1C, and the green ^31^P ssNMR spectrum in Figure 1A are recorded for InP QDs that have been treated with InF_3_ for 60 min at 180 °C, which we found most effective in increasing the PLQY. We will return to these results below, after we have discussed the screening of various ligand treatments and the optimization of the experimental conditions during the treatment.

A series of metal salts was tested as Z-type ligands to select the ligand that results in InP QDs with the optimal PLQY and fwhm. Based on previous studies on the addition of Z-type ligands to boost the PLQY of QDs, we chose to test metal halides as ligands.^39,42^ Metal halides are relatively small compared to metal carboxylates and should therefore reduce steric hindrance and enable a high surface coverage of Z-type ligands.^39^ The metal halides tested for this study are InCl_3_, ZnCl_2_, ZnF_2_, ZnBr_2_, ZnI_2_, AlF_3_, AlCl_3_, CdCl_2_, MgF_2_, and InF_3_; the QDs are exposed to these metal halides for 30 min at 150 °C.

Typical ligand treatments with metal halides use primary amines (usually oleylamine), that act as L-type ligands and solubilize both the metal halide salt as well as the QDs after ligand treatment.^39−41^ Motivated by the high PLQY obtained after the *in situ* HF treatment reported in the work of Ubbink *et al.*, which did not include any amines, we chose here to work with the pure metal halide salts.^18^ However, metal halides are small and polar compared to long carboxylic acid chains. Therefore, a complete coverage of the InP QD surface with metal halides after the treatment leads to an unstable dispersion of the QDs in hexadecane. To prevent precipitation, In(PA)_3_ is added to the treatment. For QDs to have a high PLQY and to be stable in dispersion, the QDs should contain In(PA)_3_ and metal halide ligands in an optimal ratio such that all surface atoms are covered, yet the QDs are still stable in dispersion.

The InP QDs treated with InCl_3_, ZnCl_2_, ZnBr_2_, ZnI_2_, AlCl_3_, and CdCl_2_ all precipitated. Efforts to redisperse these QDs in more polar solvents were unsuccessful. Treatments using ZnF_2_, AlF_3_, MgF_2_, and InF_3_ on the other hand resulted in QDs that from stable dispersions in hexadecane. This shows that, under these circumstances, only QDs treated with metal fluoride salts are colloidally stable in hexadecane.

The surface treatment with metal halide salts (MX_*n*_) can be seen as the following, simplified reaction, wherein MX_*n*_ competes with In(PA)_3_ for surface sites
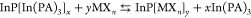
1

The fact that the treatment with the nonfluoride metal halides results in a loss of colloidal stability can be explained based on the solubility of the ligands. The water solubility values of the metal halides used in this study are shown in Table S1 in the Supporting Information. The solubility of the fluoride salts is, in general, a factor 10–100 lower than nonfluoride salts. This is largely caused by the larger lattice free energy of fluoride salts which in turn is the result of the small ionic radius of the fluoride ion. So, while the reported solubilities relate to water as a solvent, a similar trend is expected in other solvents. A higher solubility of the salts results in a higher concentration of the metal halides during the treatment. This higher concentration will shift the equilibrium in Reaction 1 to the right, resulting in an almost complete coverage of the surface with metal halides. The fact that the outcome of the treatment depends on the equilibrium between the In(PA)_3_ and metal halide ligands on the surface indicates that the other metal halides could be successful at passivating the surface of the QD, provided that the right ratio of metal halide to In(PA)_3_ is found, or if amines are added to act as L-type ligands on the InP QD surface. In this work, however, we will focus on the fluoride metal halides.

**Figure 2:**
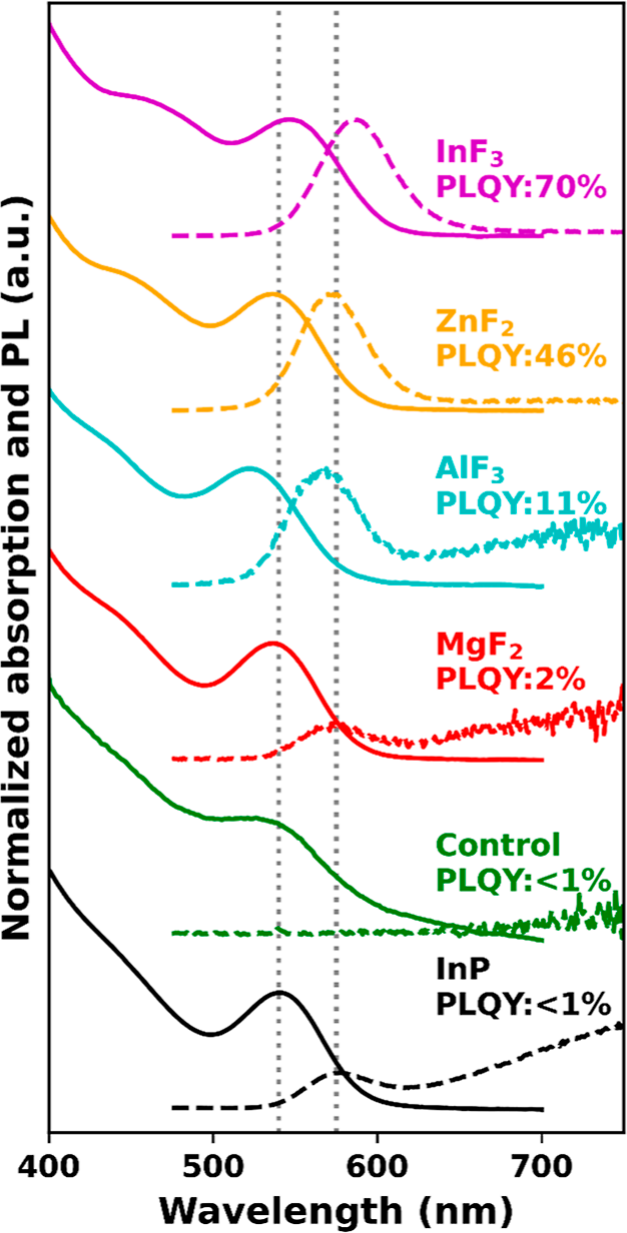
Absorption (solid) and PL (dashed) spectra of InP before (black) and after treatment at 150 °C for 30 min with various metal halide salts: MgF_2_ (red), AlF_3_ (turquoise), ZnF_2_ (orange), and InF_3_ (purple). The PLQY before and after the treatment is shown in the figure. The gray dashed lines indicate the wavelength of the 1S absorption and emission peaks before treatment.

Figure 2 displays the absorption (solid) and PL spectra (dashed) of InP QDs before and after treatment with various metal fluorides at 150 °C for 30 min. The 1S transition in absorption and PL of the untreated QDs are, respectively, observed at 540 and 575 nm and are indicated with dashed gray lines. The spectra of a control treatment with In(PA)_3_ but without a metal fluoride at 150 °C for 30 min show a widening of the 1S peak indicative of size broadening. The PLQY remained <1%, indicating that the In(PA)_3_ treatment does not effectively passivate surface defects.

The red, turquoise, orange, and purple spectra show the absorption and PL after exposing the QDs to MgF_2_, AlF_3_, ZnF_2_, and InF_3_, respectively. The treatment with MgF_2_ raised the PLQY to 2% and AlF_3_ improved the PLQY to 11%. The AlF_3_ treatment led to a blue-shift of the absorption and PL spectra, indicating a decrease in the effective size of the InP QDs. The decrease in the size could be the consequence of replacement of outer In^3+^ ions with Al^3+^ atoms, effectively reducing the size of the InP core and suggesting an exchange of In(PA)_3_ with AlF_3_, as indicated in Reaction 1. The treatment with ZnF_2_ resulted in a PLQY of 46%, and both the absorption and PL peaks are slightly shifted to shorter wavelengths, again indicating a decrease in size of the QDs. The highest PLQY, 70%, is obtained by treatment of the InP QDs with InF_3_. In this case, a small red-shift is observed in the absorption and PL spectra, suggesting that the addition of InF_3_ results in a net size increase, hence more In on the surface. Thus, in addition to replacing In(PA)_3_ with InF_3_, the surface coverage of Z-type InF_3_ ligands has increased, implying that *y* > *x* in Reaction 1. The use of InF_3_ to passivate the surface of InP QDs is similar to what was reported previously by Ubbink *et al.* but an important difference is the addition of In(PA)_3_.^18^ As mentioned earlier, In(PA)_3_ ensures that InP QDs with a high coverage number remain stable in dispersion.

A slight broadening of the absorption and PL line widths, indicative of etching, is observed but this broadening is rather small compared to HF-based treatments reported in the literature.^18,20,34^ We will come back to this issue of etching below. InF_3_ is thus selected as the most promising Z-type ligand for surface passivation of InP QDs. As we will show next, the procedure can be significantly improved by a further optimization of the reaction conditions.

### Optimization of the InF_3_ Treatment

**Figure 3:**
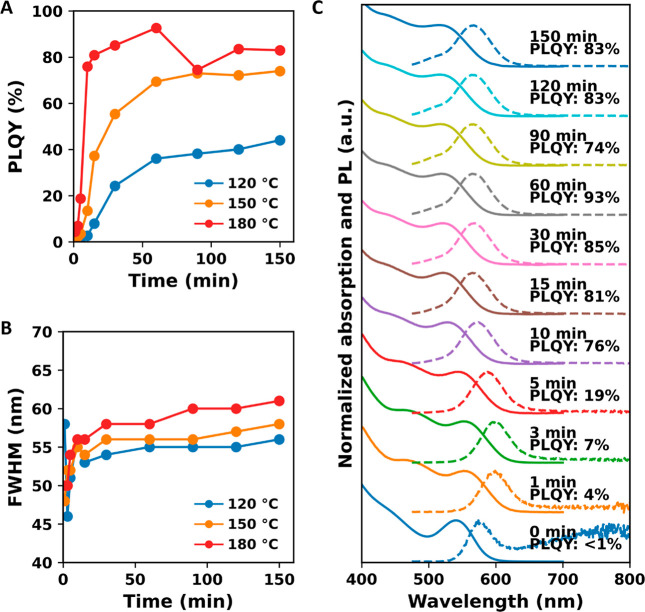
Optimization of the InF_3_ treatment. (A) PLQY and (B) fwhm of InP QDs as a function of the treatment time and temperature. (C) Absorption and PL spectra of aliquots collected during the treatment at 180 °C. Time and PLQY are shown on top of each plot.

The treatment of InP QDs with InF_3_ was optimized by measuring the optical properties of the InP–InF_3_ QDs after treatment at 120, 150, or 180 °C at different time intervals. Figure 3A shows the PLQY as a function of the treatment time for these three temperatures. For all three temperatures, three phases are observed during the treatment. In the first 30 min, a fast increase in PLQY is observed. In this initial phase, the PLQY increases from <1 to 24% at 120 °C, to 55% at 150 °C, and to 85% at 180 °C. From these results, it is clear that the chemical reaction that is happening in this first phase is thermally activated.

In the second phase, in the subsequent 30 min, a further but slower increase in PLQY is observed for all three temperatures. After 60 min, the third phase occurs where a plateau in the PLQY is reached for the treatment at 120 and 150 °C, and a slight decrease is observed for the treatment at 180 °C. The highest PLQYs obtained are 44, 74, and 93% for the treatments at 120, 150, and 180 °C, respectively. For the treatment at 120 and 150 °C, the maximal PLQY is reached after 150 min; at 180 °C, the maximum PLQY is obtained after 60 min. These results show that both the rate of PLQY increase and the final PLQY depend on temperature, suggesting that the exchange Reaction 1 is thermally activated, and perhaps is endothermic, such that the equilibrium shifts to the right with increasing temperature.

Figure 3B shows the fwhm of the PL peak as a function of the treatment time for the three treatment temperatures. Regardless of the temperature, we observe an initial rise in fwhm in the first 10 min, followed by a much slower and smaller increase. The fwhm increase is smallest for the treatment at 120 °C and largest at 180 °C, demonstrating that the fwhm increase is related to temperature. The broadening of the optical features is also visible in the control experiment, as shown in Figure 2, where InP QDs are heated in the presence of In(PA)_3_ without a metal halide present. This suggests that the process is simply induced by the prolonged exposure to elevated temperatures, and not to the InF_3_ treatment, for example due to Ostwald ripening.

From the development of the PLQY with reaction time, it is clear that the highest PLQY can be obtained with the treatment at 180 °C, at the expense of a limited increase in the fwhm of the PL. Considering both criteria, the treatment at 180 °C for 60 min provides the best balance between a PLQY increase and a limited increase in fwhm, prompting a PLQY increase up to 93% and a fwhm of 58 nm. To the best of our knowledge, this is the highest PLQY reported for core-only InP QDs.

To study the reproducibility of the treatment and the error on our PLQY measurement, we treated five InP QD samples using the optical conditions identified above. Table S2 in the Supporting Information displays the PLQY of these five samples measured two times with a calibrated integrating sphere and two times with a reference dye. On average, a PLQY of 91.6 ± 3.2% was measured using the reference dye, and a PLQY of 89.9 ± 3.5% was obtained with the integrating sphere.

### Analysis of Optical and Structural Changes during the InF_3_ Treatment

The absorption and PL spectra of InF_3_-treated InP QDs (green lines) are compared to the as-synthesized InP QDs (black lines) in Figure 1B. After the InF_3_ treatment, the PL decay, as shown in Figure 1C, is fitted with a biexponential function [0.30 exp(−*t*/42 ns) + 0.70 exp(−*t*/108 ns)] corresponding to an average PL lifetime of 99 ns. This lifetime is significantly longer than that observed for CdSe and CdTe QDs with near-unity PLQY^39,41^ but is similar to the PL lifetime of high PLQY InP prepared *via* the *in situ* HF treatment we reported previously.^18,46^

We also investigated the presence of oxides on the surface of the InP QDs with ssNMR. In Figure 1A, the one pulse ^31^P NMR spectrum is shown in green for the InP–InF_3_ QDs. The nature of the surface oxides, PO_4_^3–^, did not change during the treatment; however, the amount of oxides slightly increased (6% after InF_3_*vs* 5% before). These results are in agreement with the work of Ubbink *et al.* which showed that mainly polyphosphates result in trap states for InP QDs, and that phosphate does not introduce trap states in the band gap.^18^

The ssNMR spectrum also shows that the amount of TOPO decreases compared to untreated particles, indicating that TOPO is replaced by InF_3_ and In(PA)_3_ during the treatment. Additionally, the phosphide resonance at ∼200 ppm moves to a more negative chemical shift after treatment. Tomaselli *et al.* showed that a reduction in size can cause such a shift for InP QDs.^48^ However, the resonance shift is much larger than what would be reasonably expected for a change in QD size. We therefore speculate that the nearby presence of fluoride influences the chemical shift of the P^3–^ resonance.

The presence of In(PA)_3_ after InF_3_ treatment is determined by comparing the ^1^H NMR spectra of the QDs with a ^1^H NMR spectrum of In(PA)_3_. The spectra in Figure S1A,B, in the Supporting Information, show resonances at 0.88, 1.25, 1.55, and 2.32 ppm for both the In(PA)_3_ and the QDs. This confirms that In(PA)_3_ is present in the QD sample. The broadening of the resonances at 1.55 and 2.32 ppm indicates that the majority of the In(PA)_3_ is coordinated to the QDs, though a small amount of free palmitate is observed.

The XPS spectrum in Figure S2A in the Supporting Information displays that after InF_3_ treatment F is present in the QD sample, while none is detected before. After treatment, we observe that the In/P ratio increases from 1.3 to 2.0 (see Table S3 in the Supporting Information). This suggests that the surface coverage of In has increased, although the ratio of 2.0 seems unrealistically high, even for the small InP QDs investigated. We consider that some of the additional In is present as excess In(PA)_3_, in line with the relative increase in C and O content. If we assume that all additional In resides on the surface and that the core has an In/P ratio of 1:1, we estimate from the atomic composition a surface InF_3_/In(PA)_3_ ratio of 1:5.5. If we assume all measured carbon is part of InPA_3_, we estimate a InF_3_/In(PA)_3_ ratio of 1:4. These numbers set a lower and upper bound to the real InF_3_/In(PA)_3_ ratio on the QD surface.

Additionally, ^19^F NMR spectra are recorded for InF_3_ and InP–InF_3_ QDs and are shown in Figure S3. The solubility of InF_3_ is low in any solvent, but it is found that DMSO-*d*_6_ dissolves a measurable amount of InF_3_. The ^19^F NMR spectrum for InF_3_ shows a doublet around −166 ppm. However, in the ^19^F NMR spectrum for InP–InF_3_, recorded in CDCl_3_ (since the QDs are insoluble in DMSO), no signal is observed. It is common for NMR signals from ligands to broaden when the ligand is coordinated to the QD surface due to the slower molecular tumbling compared to the free molecules in solution.^50,51^ Since it is clear from the XPS measurements that F is present in the InP–InF_3_ samples, this suggest that all F resides on the QD surface and that ^19^F NMR spectrum of the InP–InF_3_ QDs has broadened so much that it becomes impossible to distinguish it from the measured noise.

It is concluded from the ssNMR, ^1^H NMR, ^19^F NMR, and the XPS that the InF_3_ treatment leads to the introduction of InF_3_ as Z-type ligand on the QD surface without the complete exchange of In(PA)_3_. Both InF_3_ and In(PA)_3_ reside on the surface. The In(PA)_3_ is responsible for the colloidal stability in nonpolar solvents, while the small InF_3_ increases the surface coverage of Z-type ligands and results in full passivation of P dangling bonds.

### Preventing Etching during InF_3_ Treatment

Figure 3C shows the absorption and PL spectra for each data point during the treatment performed at 180 °C. After an initial red-shift, indicating addition of InF_3_ to the surface, a blue-shift is observed for the 1S peak for longer treatment times, both in absorption and PL. Additionally, a shoulder appears on the blue side of the PL spectrum and becomes more pronounced at prolonged treatment times. The absorption and PL spectra of QDs treated at 120 and 150 °C are shown in Figure S4A,B and display similar blue-shifts and the rising of a PL shoulder at shorter wavelengths during the treatment. The shoulder at the blue sides of the PL spectra contributes to the increase in fwhm observed during the treatment. We found that this blue-shift is the result of the presence of trace amounts of free Brønsted acids which etch the QDs.

**Figure 4:**
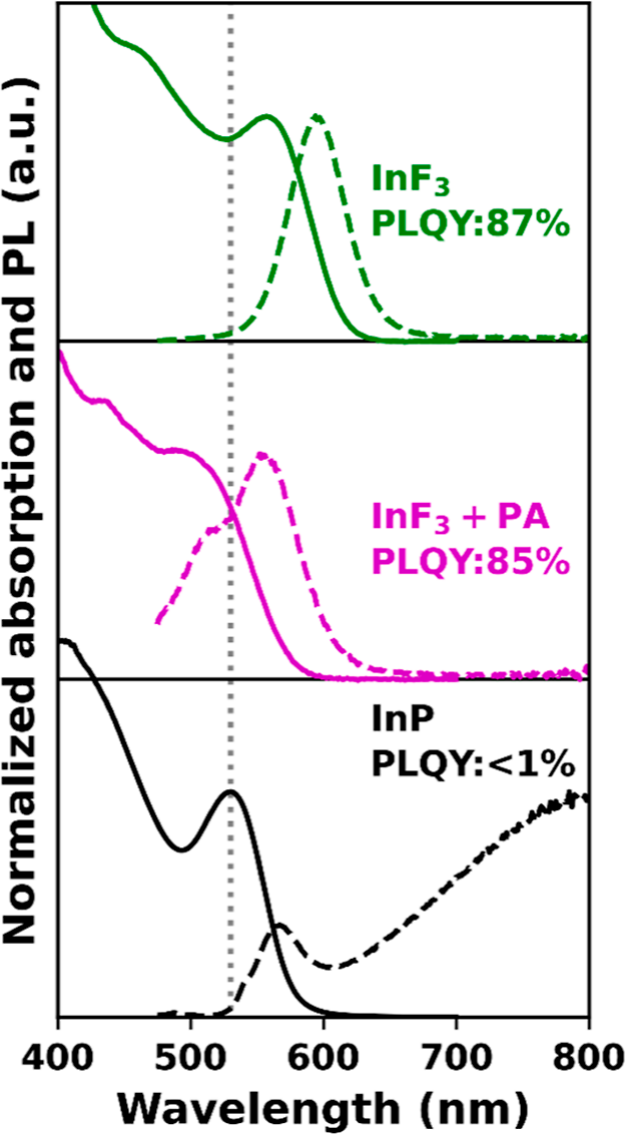
Absorption (solid) and PL (dashed) spectra for InP QDs before (black) and after InF_3_ treatment at 180 °C for 60 min under excess acid (purple) and acid-free (green) conditions. The dashed gray line indicates the 1S absorption peak for the InP QDs before treatment.

To test the effect of free Brønsted acid during the InF_3_ treatment, an InF_3_ treatment was performed both under strict acid-free conditions and in the presence of excess palmitic acid. The resulting absorption and PL spectra are shown in Figure 4 together with the spectra for the InP QDs before treatment in green, purple, and black, respectively. For the InF_3_ treatment in the presence of excess acid, the absorption blue-shifts and broadens, while a red-shift is observed after an acid-free treatment. The red-shift is in agreement with the increased size observed in TEM images, 2.9 nm before and 3.3 nm after treatment, as shown in Figure S5 in the Supporting Information. Additionally, a clear blue-shifted shoulder is observed in the PL spectrum for the treatment with excess acid, and the fwhm of the PL increases to 87 nm, *vs* 50 nm for the acid-free treatment. The PLQYs obtained for the treatment with and without acid are 85 and 87%, respectively, showing that the presence of acid does not significantly affect the PLQY. Overall, this suggests that a small amount of Brønsted acid does not result in a different surface composition, but that it does cause deleterious etching of the InP QDs, similar to what was observed for HF treatments.^18,29,31,32^ We attribute the blue-shifted shoulder in the PL spectrum to a fraction of smaller QDs generated *via* acid etching.

We note that avoiding this etching requires stringent acid-free conditions. In our case, this included the use of clean, anhydrous solvents, pure In(PA)_3_ without any free palmitic acid and, surprisingly, the use of clean and new stir bars. The acid-free nature of the In(PA)_3_ was confirmed by ^1^H NMR where an acid proton, at 10.88 ppm, as observed for PA is absent, see Figure S6 in the Supporting Information. Proposedly, the PTFE coating of stir bars gets damaged at high temperatures and is prone to absorbing and releasing contaminants, an observation that has been reported before.^52^

### Generality of the Method

**Figure 5:**
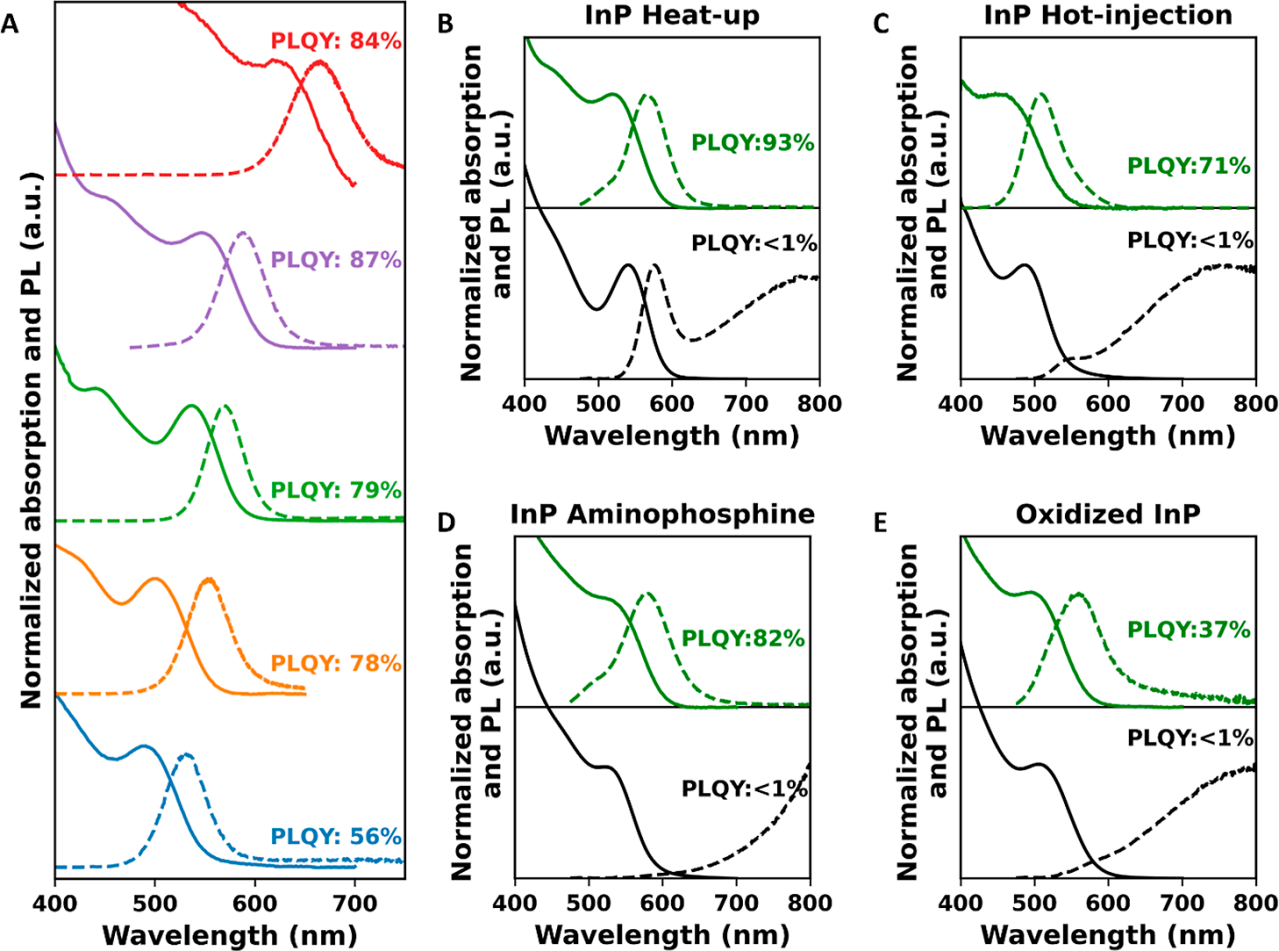
(A) Absorption (solid) and PL (dashed) spectra after InF_3_ treatment at 180 °C for 60 min for five sizes of InP QDs. (B–E) Absorption (solid) and PL (dashed) spectra before (black) and after (green) InF_3_ treatment at 180 °C for 60 min for InP QDs made *via* the heat-up synthesis method, InP QDs made *via* the hot-injection method, and InP QDs made with the aminophosphine precursors and that are oxidized, respectively.

We tested the generality of the method by performing the InF_3_ treatment on InP QDs of different sizes and on InP QDs and made *via* different synthesis routes. We note that the treatment was not optimized for each case. The InF_3_ treatment is applied to five different sizes of InP QDs with the exciton absorption, ranging from 490 to 630 nm. The QDs with the exciton absorption at 490, 540, and 560 nm were synthesized *via* a heat-up method, and those with an exciton absorption at 500 and 630 nm *via* a hot-injection synthesis. The absorption and PL spectra for these five InP QDs sizes after InF_3_ treatment are shown in Figure 5A together with their PLQY.

The PLQY increased from <1% to over 55% for the smallest InP QDs and peaks above 75% for the three other sizes of QDs. The PLQY has not been optimized independently for each QD size, which probably explains why the PLQY is not as high as that shown in Figure 2, but it is evident that the treatment is effective on all sizes.

Finally, we tested whether the effectiveness of the InF_3_ treatment depends on the synthesis method and consequently the surface composition. In the literature, two types of phosphorus precursors are commonly used in the synthesis of InP QDs: tris(trimethylsilyl)phosphine (TMSP) or aminophosphines such as tris(dimethylamino)phosphine and tris(diethylamino)phosphine.^15,26^ In general, the synthesis with TMSP is performed at higher temperatures and involves long chain carboxylic acid ligands.^6,15,44,46^ The synthesis with aminophosphines, on the other hand, is performed at lower temperatures and is often combined with halide salts and fatty amine ligands.^26,53^ One of the main differences between InP QDs made *via* these two methods is therefore the ligands that are passivating the surface of the QDs. In the case of the synthesis with aminophosphines, this results in a surface copassivated by halides and amines, whereas a synthesis with TMSP generally leads to surface passivated with long carboxylic acids.^6,25,54^ Within the TMSP synthesis method, two different methods can be distinguished: the hot-injection synthesis and the heat-up synthesis method. The difference between these synthesis methods is the temperature when the phosphorus precursor is injected, which as mentioned earlier, results in changes in the degree of surface oxidation.^18^

InP QDs made *via* the heat-up synthesis, the hot-injection method and with the aminophosphine precursor are synthesized, as described in the Methods/Experimental Section. Furthermore, to test whether the InF_3_ treatment is also suitable for boosting the PLQY of InP QDs with a high degree of surface oxidation, InP QDs were deliberately oxidized after synthesis. The oxidation is performed by exposing the InP QDs made *via* the heat-up synthesis to an atmosphere of 20% O_2_ and 80% N_2_ gas at 120 °C for 30 min.

The InF_3_ treatment was performed on the four types of InP QDs. The absorption and PL spectra are shown as the green lines in Figure 5B–E, and the PLQY is indicated in the figures. For all QDs, there is a clear increase in PLQY, but the final PLQY differs. The InP QDs made *via* the heat-up synthesis reach 93% PLQY, while those made *via* the hot-injection method reach 71%. The InP QDs synthesized with aminophosphine precursors reach a PLQY of 82%. This PLQY is similar to the results obtained by Reiss and co-workers after performing an *in situ* HF treatment on InP QDs made with aminophosphine precursors.^36^ An important difference, however, is that their result is obtained at room temperature, whereas the InF_3_ in this work is performed at 180 °C. We remark again that the full optimization of the InF_3_ treatment was performed only for the QDs made with the heat-up synthesis, and these conditions were applied to all samples. Hence, it is possible that higher PLQY values can be achieved for the other synthesis methods if the conditions are optimized. We interpret these results to mean that the InF_3_ treatment is applicable to InP QDs regardless of their synthesis method or the surface composition.

In contrast, the maximum achieved PLQY for the InP QDs that were oxidized on purpose was only 36%. This suggests that the degree of oxidation of the InP QD surface is crucial for the success of this treatment, even if it is clear from DFT calculations^18^ and the presence of PO_4_^3–^ in near-unity PLQY InP QD samples that surface phosphate does not introduce in-gap trap states. Rather, we speculate that too severe oxidation of the surface prevents the complete coverage of the QD surface with Z-type ligands.

## Conclusions

In conclusion, this work describes a simple but effective postsynthesis treatment that consist of covering the surface of InP QDs with InF_3_. Under optimized conditions, InP core-only QDs are obtained with a near-unity PLQY and single exponential photoluminescence decay curves. We further show that etching of the InP QDs can be fully prevent by working under acid-free conditions allowing for a near-unity PLQY without significant spectral broadening. Furthermore, it is shown that this method is effective on InP QDs of a wide range of sizes and made *via* different synthesis methods. Only severe oxidation of the surface of InP QDs limits the effectiveness of the InF_3_ treatment.

## Methods/Experimental Section

### Materials

The following materials were purchased from Merck Sigma and used as received: indium acetate [In(OAc)_3_, 99.99%], myristic acid (MA, >99%), anhydrous hexadecane (99%), trioctylphosphide (TOP, 97%), anhydrous acetone (99.8%), palmitic acid (PA, 99%), fluorescein, InCl_3_ (99.999%), ZnCl_2_ (>98%), ZnBr_2_ (99.999), ZnI_2_ (98%), AlF_3_ (99%), AlCl_3_ (99.99%), CdCl_2_ (99.99%), MgF_2_ (99.99), and tris(diethylamino)phosphine (97%). Octadecene (ODE, 90%, Merck Sigma) is degassed *in vacuo* at 100 °C before being stored in the glovebox. Ar (6 N), Ar/H_2_ (98:2, 6 N), and N_2_/O_2_ (80:20, 6 N) were purchased from Linde. InF_3_ (99.95%) and anhydrous toluene (99.8) were purchased from Alfa Aesar. NaOH pellets (98.5%) and oleylamine (80–90%) were bought from Acros. Tris(trimethylsilyl)phosphine (TMSP, 98%) was obtained from Strem. ZnF_2_ (99%), heptane (99%), chloroform, and ethanol were purchased from VWR.

### Heat-Up TMSP-Based Synthesis of InP QDs

The synthesis is adapted from method described in the work of Li *et al.*(44) In a typical synthesis, In(OAc)_3_ (200 mg, 0.685 mmol), MA (469.6 mg, 2.06 mmol), and anhydrous hexadecane (24.6 mL) were added to a three-neck round-bottom flask. The mixture was degassed at a Schlenk line under vacuum at room temperature for 30 min. Ar/H_2_ (98:2) was then bubbled through the solution at a rate of 0.3 L/min, and the mixture was heated to 150 °C for 30 min. TOP (3000 mg, 8.09 mmol) was injected into the mixture, and the mixture was reheated to 150 °C. At this temperature, a solution of TMSP (82.70 mg, 0.33 mmol) in anhydrous hexadecane (5.09 mL) was swiftly injected in the reaction mixture, and the temperature was ramped to 270 °C in 10 min. The mixture was cooled to room temperature with an air gun after the reaction had run for 7 min. The QDs were purified by precipitation with 5 volume equivalents of anhydrous acetone, followed by centrifugation at 5000 rpm for 10 min. The supernatant was discarded, and the solid residue was redispersed in anhydrous toluene. This purification step was repeated once, and afterward, the QDs were dispersed in anhydrous hexadecane.

### Hot-Injection TMSP-Based Synthesis of InP QDs

The synthesis method is based on the studies reported by Won *et al.* and Ubbink *et al.*(6,18) In a three-neck round-bottom flask, In(OAc)_3_ (585 mg, 2.00 mmol), PA (1535 mg, 6.00 mmol), and ODE (50 mL) are loaded. The flask is connected to a Schlenk line, and the mixture is degassed at 120 °C under vacuum for 60 min. The atmosphere is then changed to N_2_ gas, and N_2_ gas is blown over the surface of the reaction mixture with a rate of 0.4 L/min. The temperature is raised to 280 °C, and a solution of TMSP (375.81 mg) in TOP (5 mL) is swiftly injected, prompting a decrease in reaction temperature. The temperature is set to 260 °C, and the reaction proceeded for 12 min. The reaction mixture is cooled to room temperature with an air gun. The purification was performed in the same way as stated above for the heat-up synthesis method.

### Oxidation of InP QDs

In a typical synthesis, anhydrous hexadecane (5 mL) is loaded in a three-neck round-bottom flask and degassed at a Schlenk line under vacuum at room temperature for 30 min. The flask is then placed under an Ar atmosphere, and InP QDs (60 nmol, in anhydrous toluene) are injected. The solution is then degassed under vacuum for 30 min. The reaction flask is then disconnected from the Schlenk line, keeping the reaction mixture under reduced pressure. A gaseous O_2_/N_2_ mixture (80:20) is bubbled through the solution until atmospheric pressure is obtained. The reaction mixture is heated to 150 °C for 30 min and then cooled to room temperature with an air gun. The purification was performed in the same way as stated above for the heat-up synthesis method.

### Aminophosphine-Based Synthesis of InP QDs

The procedure is based on the method previously published by Tessier *et al.*(53) 100 mg (0.45 mmol) of indium(III) chloride and 300 mg (2.20 mmol) of zinc(II) chloride were mixed in 3 mL (9.10 mmol) of anhydrous oleylamine in a 25 mL flask. The mixture was stirred and degassed at 120 °C for an hour and then heated to 180 °C under an inert atmosphere. Upon reaching 180 °C, 0.50 mL (1.83 mmol) of tris(diethylamino)phosphine, transaminated with 2 mL (6.07 mmol) of anhydrous oleylamine, was quickly injected in the reaction mixture described above, and the InP nanocrystal synthesis proceeded for 30 min. The synthesized InP QDs were purified using anhydrous ethanol.

### Preparation of the In(PA)_3_ Precursor

The procedure is based on the method previously published in the work of Angelé *et al.*(55) PA (5.1 g, 13.2 mmol) and In(OAc)_3_ (0.6 g, 1.36 mmol) are loaded in a 25 mL three-neck round-bottom flask. The reaction mixture is stirred under reduced atmosphere at 120 °C for 6 h. The reaction mixture is then filtered over a glass filter, and the solid is washed with ethanol (5 × 20 mL), heptane (5 × 20 mL), and chloroform (1 × 20 mL). The solid is then dried *in vacuo* and subsequently stored in the glovebox.

### Metal Halide Treatment of InP QDs

In a typical metal halide treatment, the metal halide (0.73 mmol), In(PA)_3_ (44 mg, 0.05 mmol), and InP QDs (1.0 mL in anhydrous hexadecane, 50 μM) are mixed in a glass vial. The mixture is heated to a temperature between 120 and 180 °C and stirred for 1–150 min. The reaction mixture is cooled to room temperature and centrifuged at 5000 rpm for 10 min to separate the remaining solid metal halide from the QDs in dispersion. The supernatant containing the InP QDs was then diluted with anhydrous acetone and centrifuged at 5000 rpm for 10 min. The supernatant was discarded, and the treated InP QDs were redispersed in anhydrous toluene.

In a typical InF_3_ treatment performed at the optimal parameters, InF_3_ (125 mg, 0.73 mmol), In(PA)_3_ (44 mg, 0.05 mmol), and InP QDs (1.25 mL in anhydrous hexadecane, 50 μM) are mixed in a glass vial. We note that InF_3_ is poorly soluble in hexadecane so that a saturated solution with noticeable white precipitate results. The mixture is heated to 180 °C and stirred for 60 min. The separation and redispersion in anhydrous toluene are performed in the same manner as described for the general metal halide treatment.

### Optical Characterization

A PerkinElmer Lambda 365 spectrometer was used for recording the UV–vis absorption spectra. An Edinburgh Instruments FLS980 spectrofluorometer with double grating monochromators for both excitation and emission paths and a 450 W xenon lamp as an excitation source were used. PLQY values were obtained with respect to the fluorescein reference dye in 0.1 M NaOH in water at room temperature. The PLQY was calculated using the following equation
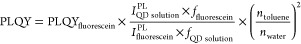
where PLQY_fluorescein_ is set to be 92% for an excitation wavelength of 465 nm,^56^*I*^PL^ is the intensity of the PL signal of either the fluorescein solution or the QD solution, *n* is the refractive index of toluene or water at 465 nm (1.4969 and 1.333), and *f* is the fraction of absorbed light for the fluorescein or toluene solution, calculated as *f* = 10^–OD^ with the OD being the optical density of the fluorescein or QD solution at 465 nm. PLQYs obtained *via* this method were found to be reproducible, with an measurement error of <3% on the PLQY values based on replication of the PLQY measurement on various samples that we prepared in identical fashion.

Additionally, the PLQY was measured using an Edinburgh Instruments FLS980 spectrometer with a calibrated integrating sphere. The emission was recorded between 475 and 800 nm, and the samples were excited at 465 nm.

An Edinburgh Instruments Lifespec TCSPC with a 400 nm pulsed laser was used for recording the PL decay traces. The PL decay traces are fitted with a biexponential or triexponential fitting function, and the intensity-weighted average lifetimes are calculated with the equation τ_ave_ = (*A*_1_τ_1_^2^ + *A*_2_τ_2_^2^)/(*A*_1_τ_1_ + *A*_2_τ_2_) and τ_ave_ = (*A*_1_τ_1_^2^ + *A*_2_τ_2_^2^ + *A*_3_τ_3_^2^)/(*A*_1_τ_1_ + *A*_2_τ_2_ + *A*_3_τ_3_) with *A*_*n*_ and τ_*n*_ as the amplitude and lifetime of the first and second exponent.

### ssNMR Characterization

For solid-state NMR analysis, samples dispersed in anhydrous toluene were dried by evaporating the solvent *in vacuo*, then the dried QDs were mixed with activated alumina and loaded into a 4 mm zirconia rotor. Measurements were performed with a Bruker Ascend 500 magnet (11.7 T) equipped with a NEO console operating at a ^31^P resonance frequency of 202.45 MHz, using a three channel DVY MAS probe from Bruker. ^31^P spectra were referenced to external H_3_PO_4_ (=0 ppm). Single pulse ^31^P spectra were collected with a MAS frequency of 8 kHz, a recycle delay (*d*_1_) of 50 s, and a 4.8 μs pulse width. Proton decoupling was performed during acquisition using the Spinal-64 decoupling sequence.

### Solution Nuclear Magnetic Resonance

An Agilent 400-MR DD2 which is equipped with a 5 mm ONE NMR probe was used to record solution NMR spectra. ^1^H NMR (399.7 MHz) spectra were obtained with a recycle delay of 1 s in deuterated chloroform. Signals are referenced with residual chloroform peaks (7.26 ppm). ^19^F NMR (399.7 MHz) spectra were obtained with a recycle delay of 1 s in deuterated chloroform or DMSO.

### X-ray Photoelectron Spectroscopy

Samples are dropcast on thin conductive substrates. XPS measurements were performed in ultra-high vacuum with a ThermoFisher K-Alpha equipped with an Al Kα source which radiates with an energy of 1486 eV. An Ar flood gun was used during the measurements to prevent charging.

### Transmission Electron Microscopy

Samples are dropcast on grids, and TEM images were acquired with a JEOL JEM1400 transmission electron microscope which operates at 120 kV.

## Supporting Information Available

Additional characterization *via*^1^H NMR, XPS, ^19^F NMR, TEM, and complete overview of optical properties of InP QD samples treated at various temperatures and times (PDF)
