# Supplementary material for: Near-Unity Photoluminescence Quantum Yield of Core-Only InP Quantum Dots via a Simple Postsynthetic InF3 Treatment
Source: ACS Nano. 2024 May 22;18(22):14685–95. doi: 10.1021/acsnano.4c03290 (PMC11155241; doi:10.1021/acsnano.4c03290)
Supplement: Supplementary file 1 — nn4c03290_si_001.pdf [file nn4c03290_si_001.pdf]

Supporting information for

Near-Unity Photoluminescence Quantum Yield of Core-Only InP Quantum Dots *via* a Simple Postsynthetic InF_3 Treatment

Maarten Stam[†]*, Guilherme Almeida*[†]*, Reinout F. Ubbink*[†]*, Lara M. van der Poll*[†]*, Yan B. Vogel*[†]*, Hua Chen*[†]*, Luca Giordano*[#]*, Pieter Schiettecatte*[#]*, Zeger Hens*[#] *and Arjan J. Houtepen*[†]*

[†] Optoelectronic Materials Section, Faculty of Applied Sciences, Delft University of Technology, Van der Maasweg 9, 2629 HZ Delft, The Netherlands

[#] Physics and Chemistry of Nanostructures, Department of Chemistry, Ghent University, 9000 Gent, Belgium

Table S1: Solubility of several metal halide salts in water at 20 °C in g/100 cm^3.[1, 2]

Compound	Formula	Solubility g/100 cm^3 in water (20 °C)
Indium(III) Fluoride	InF_3	11.2
Indium(III) Chloride	$InCl_3$	212
Zinc(II) Chloride	$ZnCl_2$	395
Zinc(II) Fluoride	ZnF_2	1.6
Zinc(II) Bromide	$ZnBr_2$	446
Zinc(II) Iodide	ZnI_2	432
Aluminum(III) Fluoride	AlF_3	0.67
Aluminum(III) Chloride	$AlCl_3$	45.8
Cadmium(II) Chloride	$CdCl_2$	135
Magnesium(II) Fluoride	MgF_2	0.007325

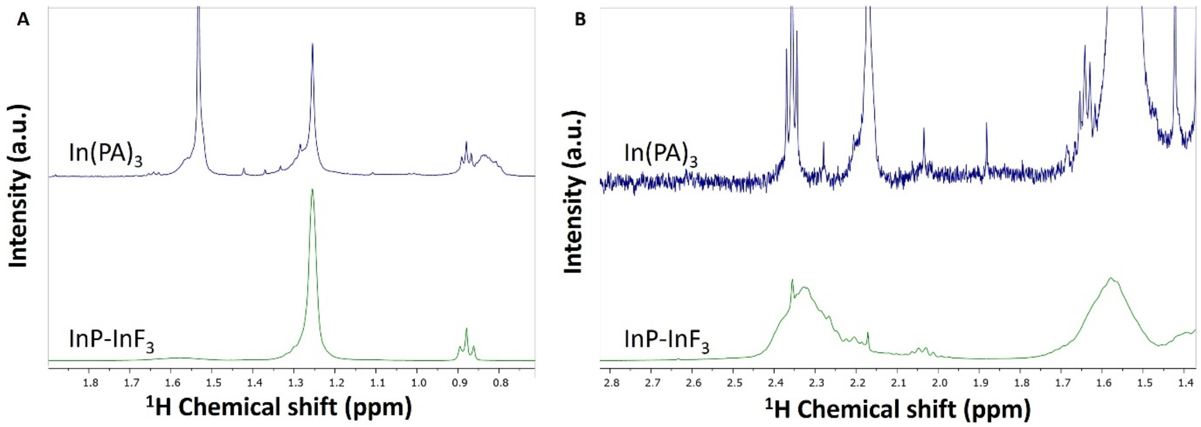

Figure S 1: ^1H NMR of $In(PA)_3$ and InP-InF_3 QDs in $CDCl_3$.

Table S2: PLQY measured for five InP QD samples treated at the optimal conditions (180 °C for 60 minutes) with respect to a reference dye and in an integrating sphere. The symbols μ and σ indicate the PLQY average and standard deviation (in absolute percentages), respectively.

Sample	PLQY ref dye (%)	PLQY ref dye (next day) (%)	PLQY sphere (%)	PLQY sphere (next day) (%)	μ dye (%)	σ dye (%)	μ sphere (%)	σ sphere (%)	μ all (%)	σ all (%)
1	91.6	89.6	83.9	88.9	90.6	1.0	86.4	2.5	88.5	2.8
2	98.5	90.3	91.6	89	94.4	4.1	90.3	1.3	92.4	3.7
3	90.6	88.4	85.4	90.2	89.5	1.1	87.8	2.4	88.7	2.1
4	94.8	87.5	87.4	93.7	91.2	3.7	90.6	3.2	90.9	3.4
5	94.9	90.1	94.2	94.4	92.5	2.4	94.3	0.1	93.4	1.9
μ	94.1	89.2	88.5	91.2	91.6		89.9		90.8	
σ	2.8	1.1	3.8	2.3	3.2		3.5		3.5	

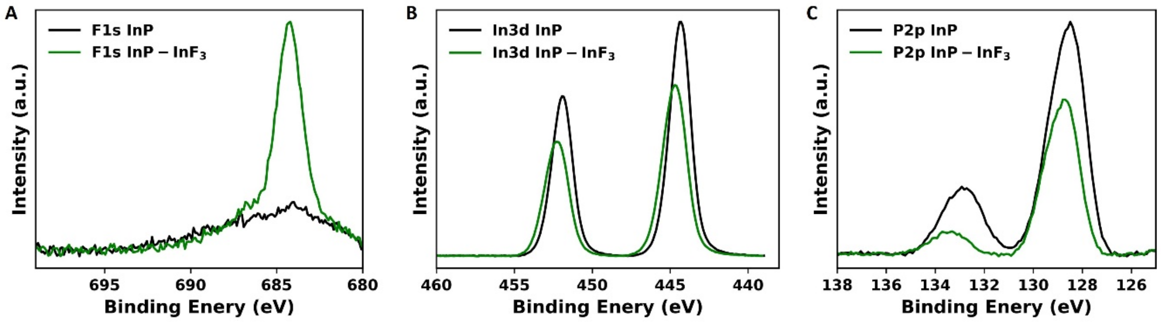

Figure S2: XPS spectrum of the F1s (A), In3d (B) and P2p (C) photoemission line for InP (black) and InP-InF_3 (green).

Table S3: Elemental composition of InP QD samples before and after InF_3 treatment, as determined by XPS.

	Surface scan		After ion beam etching	
Element	As synthesized	InF3 treated	As synthesized	InF3 treated
C(%)	75.86	74.67	72.59	82.09
In(%)	5.62	4.53	12.77	7.27
O(%)	14.33	17.51	6.58	5.76
P(%)	4.19	2.22	8.06	3.3
F(%)		1.07		1.57
In:P	1.34	2.04	1.58	2.20
In:F		4.23		4.63
P:F		2.07		2.10

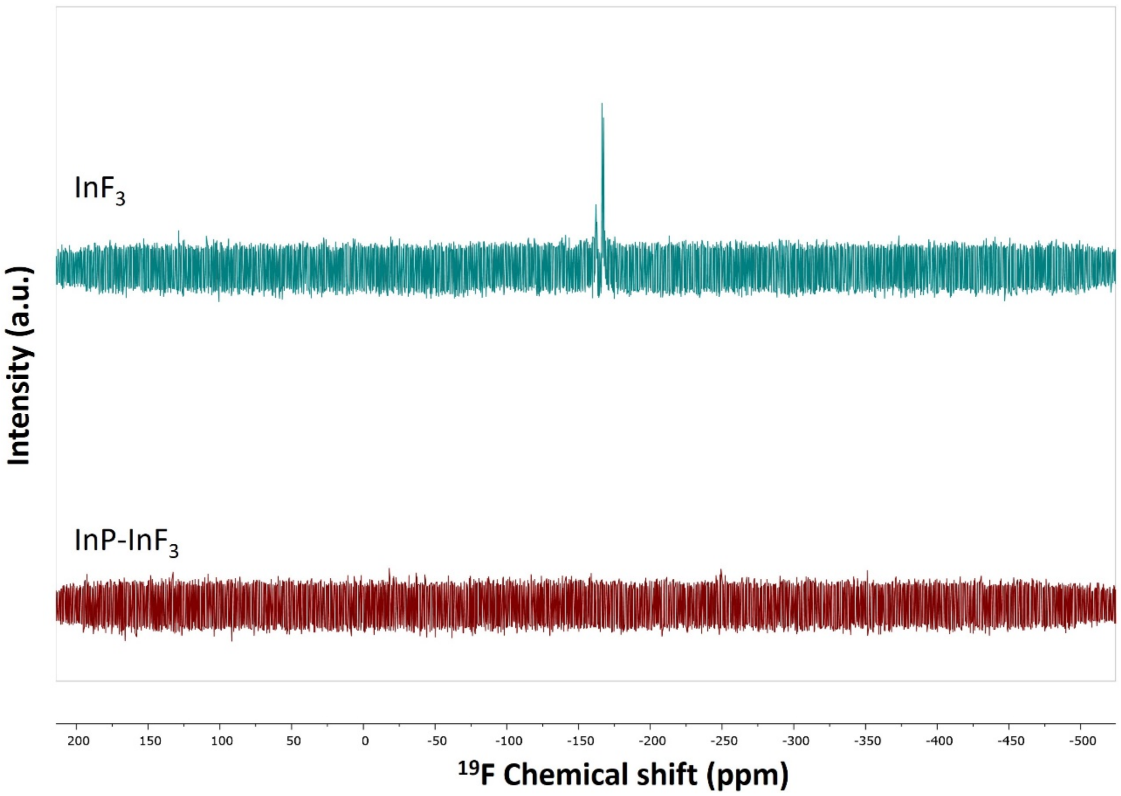

Figure S3: ^{19}F NMR of InF_3 (TOP) and $InP\text{-}InF_3$ (bottom) in DMSO-d_6 and $CDCl_3$, respectively.

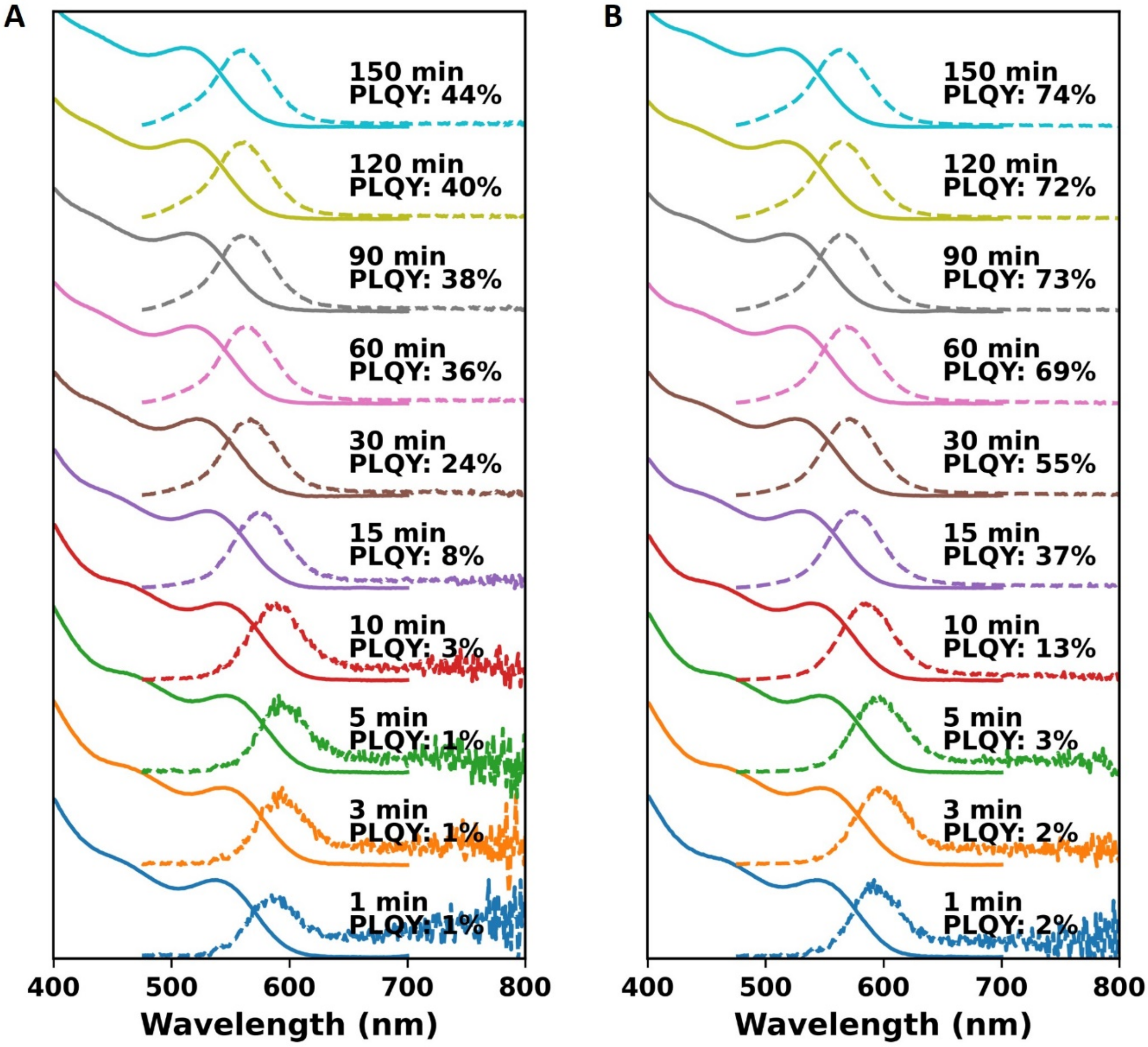

Figure S4: The absorption and PL spectra of aliquots collected during the treatment at 120 °C A) and 150 °C B). Time and PLQY are shown on top of each plot.

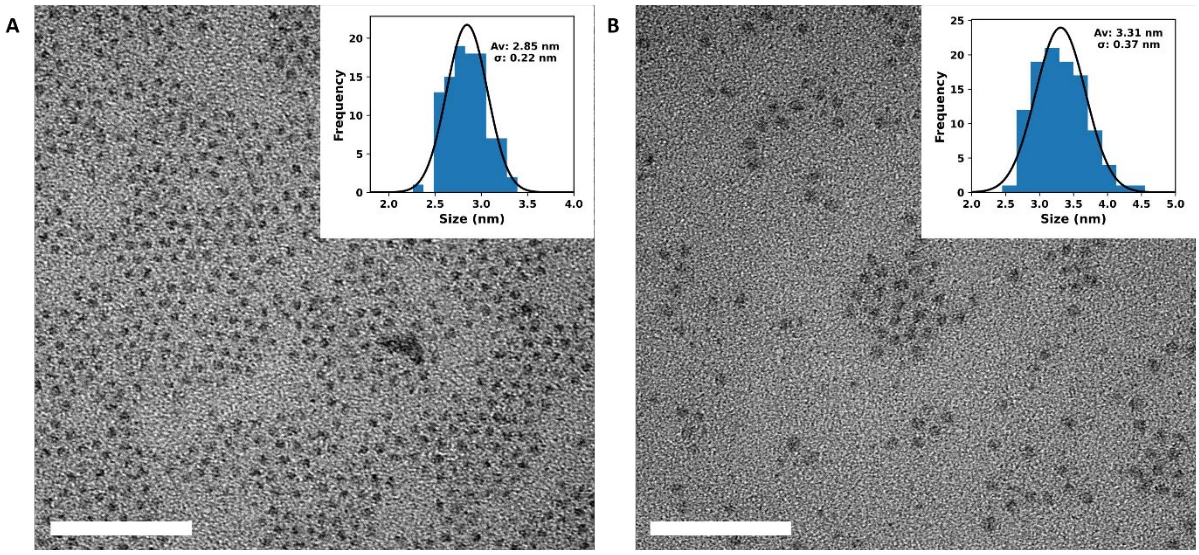

Figure S5: TEM images of A) InP and B) InP-InF_3 QDs.

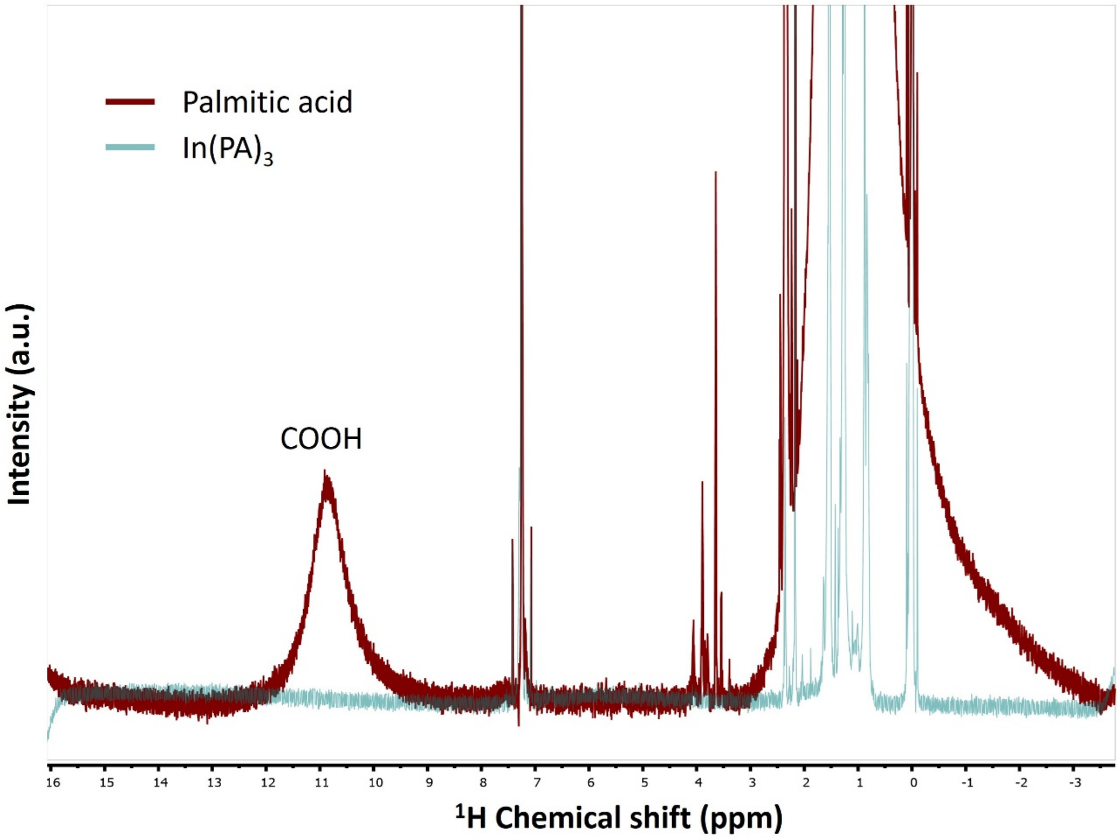

Figure S 6: ^{1}H NMR of palmitic acid (red) and $In(PA)_3$ (blue) in $CDCl_3$.

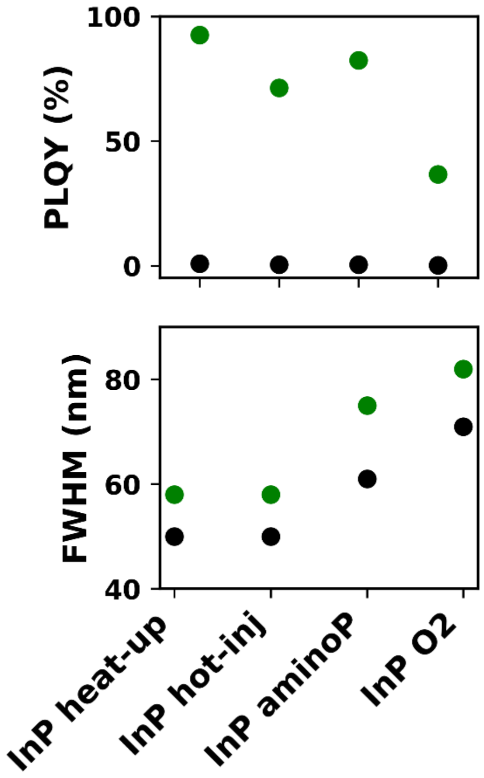

Figure S7: PLQY and FWHM for four types of InP QDs before (black) and after (green) InF_3 treatment.

REFERENCES

1. https://www.chemspider.com/, accessed on March 1st 2024.
2. https://www.chemeurope.com/en/encyclopedia/Solubility_table.html, accessed on March 1st 2024.
